# Biological lipid membranes for on-demand, wireless drug delivery from thin, bioresorbable electronic implants

**DOI:** 10.1038/am.2015.114

**Published:** 2015-11-27

**Authors:** Chi Hwan Lee, Hojun Kim, Daniel V Harburg, Gayoung Park, Yinji Ma, Taisong Pan, Jae Soon Kim, Na Yeon Lee, Bong Hoon Kim, Kyung-In Jang, Seung-Kyun Kang, Yonggang Huang, Jeongmin Kim, Kyung-Mi Lee, Cecilia Leal, John A Rogers

**Affiliations:** 1 Weldon School of Biomedical Engineering and School of Mechanical Engineering and The Center for Implantable Devices and Birck Nanotechnology Center, Purdue University, West Lafayette, IN, USA; 2 Department of Materials Science and Engineering and Frederick Seitz Materials Research Laboratory, University of Illinois at Urbana-Champaign, Urbana, IL, USA; 3 Department of Materials Science and Engineering, University of Illinois at Urbana-Champaign, Urbana, IL, USA; 4 Department of Biomicrosystem Technology, Korea University, Seoul, Republic of Korea; 5 Department of Biochemistry and Molecular Biology, Global Research Laboratory, Korea University College of Medicine, Seoul, Republic of Korea; 6 Department of Civil and Environmental Engineering, and Mechanical Engineering, Center for Engineering and Health and Skin Disease Resesarch Center, Northwestern University, Evanston, IL, USA; 7 Center for Mechanics and Materials, Tsinghua University, Beijing, China; 8 State Key Laboratory of Electronic Thin Films and Integrated Devices, University of Electronic Science and Technology of China, Sichuan, China; 9 Department of Chemistry, University of Illinois at Urbana-Champaign, Urbana, IL, USA; 10 Department of Bioengineering, University of Illinois at Urbana-Champaign, Urbana, IL, USA

## Abstract

On-demand, localized release of drugs in precisely controlled, patient-specific time sequences represents an ideal scenario for pharmacological treatment of various forms of hormone imbalances, malignant cancers, osteoporosis, diabetic conditions and others. We present a wirelessly operated, implantable drug delivery system that offers such capabilities in a form that undergoes complete bioresorption after an engineered functional period, thereby obviating the need for surgical extraction. The device architecture combines thermally actuated lipid membranes embedded with multiple types of drugs, configured in spatial arrays and co-located with individually addressable, wireless elements for Joule heating. The result provides the ability for externally triggered, precision dosage of drugs with high levels of control and negligible unwanted leakage, all without the need for surgical removal. *In vitro* and *in vivo* investigations reveal all of the underlying operational and materials aspects, as well as the basic efficacy and biocompatibility of these systems.

## INTRODUCTION

Macroscale drug delivery devices offer advantages over systemic particulate approaches with respect to target efficiency, nuclease degradation and renal clearance.^1^ Most such devices rely on passive drug diffusion from non-degradable polymer materials or release from matrices that degrade over time.^2–5^ In both cases, the engineering designs, the materials compositions and the physiological conditions pre-define the release kinetics, such that control after implantation is not possible. Precise, adjustable and patient-specific operation can be achieved with electronically programmable systems that exploit remotely triggered opening of valves built into combined fluidic and electronic platforms.^6–8^ A key disadvantage is that surgical procedures must be used to extract the implanted hardware after completion of the delivery function. Alternative strategies include those that use lipid-based materials as hosts for drugs such as doxorubicin, where *ex situ* hyperthermic treatments based on radio frequency ablation,^9^ microwaves^10^ or focused ultrasound,^11^ can trigger thermally activated release. When used in oncological applications in the liver, breast, brain or pancreas,^12^ this scheme can extend the tumor treatment zone down to microscale metastases, which are the main culprit in cancer recurrence.^13^ Such *ex situ* triggering approaches can, however, be difficult to localize precisely; they can induce ancillary tissue damage; and they are only applicable to a single type of drug. Our recent work^14^ demonstrated bioresorbable systems for *in situ* operation, but with only single-channel control over a single type of drug from a single reservoir, where matrices of silk fibroin allowed adjustment of release rates across a narrow range above a fixed, intrinsic baseline value.

This paper reports an important advance that follows from the combined use of temperature-sensitive lipid-based layered films with electronically programmable, frequency-multiplexed wireless hardware. The result allows *in situ*, on-demand release of single or multiple classes of drugs from multiple, independently controlled reservoirs, in a completely bioresorbable platform, where reversible control of the release kinetics with near-zero leakage in the off-state. Systematic *in vivo* and *in vitro* studies demonstrate the underlying principles and all of the relevant features of operation.

## MATERIALS AND METHODS

### Fabrication and device design

The device configuration allows wireless power delivery through inductively coupled coils to resistive heating elements designed to increase the temperature within a lipid membrane. Electronic components, such as inductive coils, resistive heaters and interconnects, consist of features formed in a thin molybdenum (Mo) foil. The fabrication began with laminating a thin metal foil (Mo; purity: 99.9%, Goodfellow, Coraopolis, PA, USA) on a sheet of adhesive plastic (3M, St Paul, MN, USA) as a temporary supporting layer. Micromachining with a laser-cutting tool (LMT-5000s Dual Laser System, Potomac, MD, USA) defined the patterns for inductive coils and resistive heating elements in the foil. Integration with a bioresorbable substrate involved bonding to a sheet of poly(lactic-co-glycolic acid) (PLGA) softened by heating at 60 °C for 1 min followed by cooling to room temperature. Peeling away the adhesive plastic sheet exposed the surfaces of the contact pads to allow mechanical removal of surface oxides. Sputter deposition of Mo (1.5 μm) through a shadow mask defined an interlayer electrical connection. Laminating additional layers of micromachined foils and PLGA sheets formed interconnects, dielectrics and encapsulation layers to complete the fabrication.

### Wireless power delivery system

An external primary transmission coil was constructed using planar printed circuit board technology. An alternating current waveform with a peak voltage of 1–10 V from a waveform generator (Agilent 33120A, Agilent Technologies, Santa Clara, CA, USA) was amplified to 10–20 V by a radio frequency power amplifier (210L, Electronics and Innovation, Rochester, NY, USA) and delivered to the transmitter coil. An inductively coupled coil received the amplified current waveform and delivered it to resistive heating elements. The inductance of the square receiver coil can be approximated by the expression:

$$L=\frac{1.27\mu n^{2}d_{\text{avg}}}{2}\left[\text{ln}\left(\frac{2.07}{\varphi }\right)+0.18\varphi +0.18\varphi^{2}\right]$$

where *n* is the number of turns, *μ* = *μ*_0_*μ*_r_ is the product of relative and absolute permeability, *d*_avg_ = (*d*_o_+*d*_i_)/2 is the average turn length where *d*_i_ and *d*_o_ are the inner and outer diameter of the coil, respectively, and *φ*=(*d*_0_ − *d*_i_)/(*d*_0_ + *d*_i_) is the fill factor for the coil. The design of the receiver coil determines the resonant frequency, thereby enabling multiple, independently controlled coils to be positioned in close proximity.

### Synthesis of lipid membrane

Lipid compounds dissolved in either a chloroform or a mixed solution of chloroform, methanol and deionized water (65:35:8 by weight) were purchased from Avanti polar lipids, Alabaster, AL, USA. The preparation began with spin casting of pre-mixed lipid stock solution (60 μl) of dipalmitoylphosphatidylcholine, 1,2-dilauroyl-sn-glycero-3-phosphoethanolamine, dipalmitoylphosphatidylglycerol or 1,2-dioleoy1-3-trimethylammonium-propane and cholesterol with a mole ratio of 35 : 35 : 10 : 20 on the surface of the device. Placing the coated device in a desiccator under vacuum (<75 torr) for overnight allowed self-assembly of the lipid into a multilayer membrane and complete removal of residual solvent. Laminating a temporary shadow mask (Mo foil, 5 μm thickness) selectively exposed the serpentine resistor. Heating the device at 55 °C for 30 s with a drop (30 μl) of solution containing a drug of interest allowed hydration and self-assembly into a liquid-disordered (L_d_) phase of the lipid membrane, thereby trapping the drug. Spinning the device twice at 2000 r. p.m. for 40 s and then immersing it in deionized water (60 ml) for more than 2 days eliminated any untrapped drugs. The deionized water was replaced every other day.

### Measurements of drugs

Amounts of released drug were measured using a plate reader (Victor 3 Multilabel Reader, Perkin-Elmer, Waltham, MA, USA). The fluorescence intensity measured relative to the standard curve (Supplementary Figure S1) was used to calculate the amounts of doxorubicin^15^ (Sigma-Aldrich, St Louis, MO, USA) and Alexa Fluor 488 conjugated Dextran (3000 MW, anionic; Life Technologies, Carlsbad, CA, USA). Amounts of the released parathyroid hormone (1–34) were measured by absorbance (optical density) through an enzyme immunoassay kit (Phoenix Pharmaceuticals Inc., Burlingame, CA, USA). Applying a drop (20 μl) of surfactant (Triton X-100, Sigma-Aldrich) destroyed the structure of the lipid membrane, allowing the complete release of the remaining drugs into surroundings.^16^ The measured amounts of the remaining drugs in the lipid membrane were insignificant, typically within ~ 0.1 μg.

### Cell culture experiments and proliferation/viability assay

Human tumor cells (HeLa) were cultured in Dulbecco’s Modified Eagle’s Medium (mixture of 10% fetal bovine serum and 1% penicillin–streptomycin) at 37 °C with 5% of carbon dioxides (CO_2_). The proliferation/viability assays followed protocols for commercially available kits (CellTiter 96 AQueous One Solution Cell Proliferation Assay (Promega, Madison, WI, USA) and Cytotox ONE (Promega) for proliferation and viability assays, respectively) with ~ 10 000 seeded cells per wells on 96-well plates (Falcon 96-Well Cell Culture Plates, Corning, Corning, NY, USA).

### *In vivo* cytotoxicity

All animal studies were performed in accordance with Institutional Animal Care and Use Committee. Balb/c mice (female, 7 weeks) were anaesthetized with 30 mg kg^−1^ zolazepam hydroxide (Zoletil 50; Virbac, Sao Paulo, Brazil) and 10 mg kg^−1^ zylazine hydroxide (Rumpun; Bayer, Shawnee Mission, KS, USA) via intraperitoneal injection. Prior to the implantation, test and reference materials (HDPE (high-density polyethylene)) were sterilized with ethylene oxide. Materials were inserted in subcutaneous pockets made on the dorsal of the animal for 5 weeks. For histological analysis, the skin surrounding the tested materials was fixed in 10% neutral-buffered formalin, embedded in paraffin, stained with hematoxylin and eosin.

### Flow cytometry analysis

The following Abs were purchased from eBioscience (San Diego, CA, USA): anti-mouse CD4 (RM4–4), Ly6G (RB6–8C5), CD3 (145–2C11), CD19 (1D3), CD49b (Dx5), CD8 (53–6.7), CD11b(M1/70) monoclonal antibodies conjugated with fluorescein isothiocyanate, phycoerythrin, phycoerythrin-cyanine dye, peridinin chlorophyll protein complex with cyanin-5.5, allophycocyanin, allophycocyanin-cyanine dye, Alexa Fluor 488. Single-cell suspensions from blood and spleen were treated with 2.4G2 anti-CD16/CD32 mAb (2.4G2, Fc block) to block FcRIII/II receptors. Cells were then incubated for 20 min at 4 °C. Multicolor analysis was performed using a FACS Canto II (BD Bioscience, San Diego, CA, USA) and analyzed with FlowJo software (TreeStar, Ashland, OR, USA).

### Measurement of cytokine by cytometric bead arrays

The levels of interleukin-10 (IL-10), IL-6, interferon-γ, tumor necrosis factor-α and IL-12p70 in the serum were measured by mouse inflammation cytometric bead arrays kit (BD Biosciences, San Diego, CA, USA).

### Statistics

The data are represented as mean ± s.e.m. Statistical significance was determined by one-way analysis of variance followed by Bonferroni’s multiple comparison test. Differences were considered significant when the *P*<0.05. All analyses were conducted using the Prism software (Graph Pad Prism 5.0, La Jolla, CA, USA).

### Finite element analysis

ABAQUS commercial software (ABAQUS analysis user’s manual V6.10, Providence, RI, USA) was used to study the thermal response of receiver coils and resistor on a substrate. Both the receiver coils and resistor (Mo, 15 μm thickness, thermal conductivity: 138 W m^−1^ K^−1^, density: 10 330 kg m^−3^, specific heat: 251 J K^−1^ kg^−1^) were modeled by the heat transfer shell element (DS4), while the substrate (PLGA, 200 μm thickness, thermal conductivity: 0.3 W m^−1^ K^−1^, density: 1580 kg m^−3^, specific heat: 1200 J K^−1^ kg^−1^)^17^ and the porcine tissue (1–3 mm, 0.45 W m^−1^ K^−1^, density: 1024 kg m^−3^, specific heat: 3350 J K^−1^ kg^−1^)^18^ were modeled by the heat transfer brick element (DC3D8).

## RESULTS AND DISCUSSION

**Figure 1:**
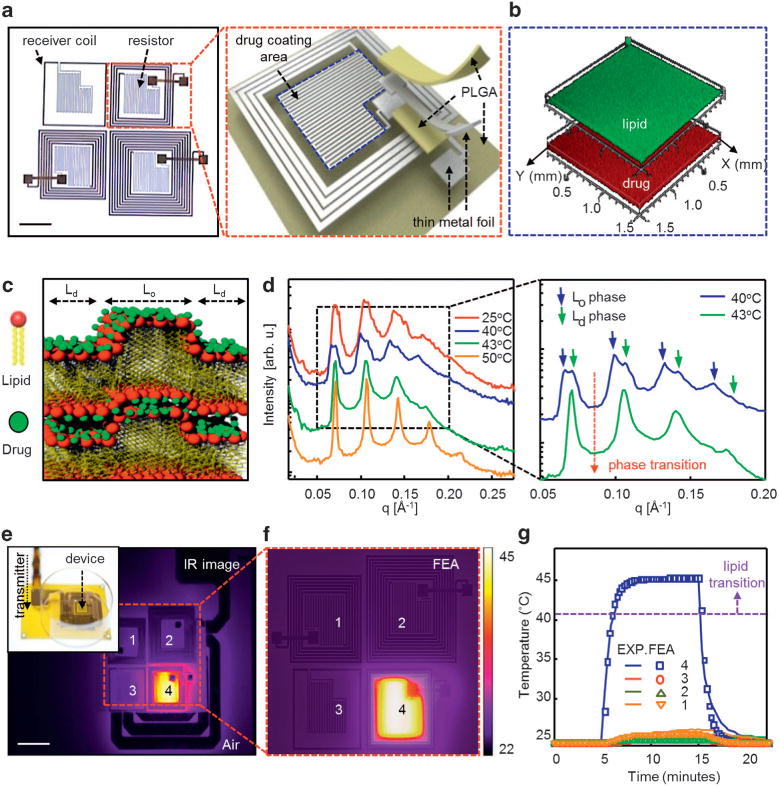
Layouts and characteristics of bioresorbable, wirelessly programmable drug delivery devices that exploit thermally activated lipid membranes. (**a**) Optical image (scale bar: 5 mm) of a device that consists of a 2 × 2 array of inductive coupling coils and serpentine thermal actuators on a bioresorbable substrate (left). Schematic exploded view illustration (right). (**b, c**) Three-dimensional (3D) Z-stacked confocal microscope images and schematic illustrations of a lipid membrane loaded with drug molecules. (**d**) Small angle X-ray scattering (SAXS) scans from a lipid membrane at temperatures between 25 and 50 °C (left), with expanded view between 40 and 43 °C (right) showing the transition from an liquid-ordered to a liquid-disordered state. (**e**) An infrared (IR) image (scale bar: 1 cm) collected during operation of a representative device with a corresponding optical image (inset) showing spatially controlled heating (**f**) Distribution of temperature determined by finite element analysis (FEA). (**g**) Experimental (lines) and FEA (dots) results for average temperatures (marked 4 in **e, f**). The temperature of the wirelessly triggered actuator reaches 45 °C while the other regions (marked 1, 2, 3 in **e, f**) remain at nearly room temperature. FEA, finite element analysis; L_d_, liquid disordered; L_o_, liquid ordered; PLGA, poly(lactic-co-glycolic acid).

Figure 1a presents an optical image and an exploded view schematic illustration of a representative system. The construction relies exclusively on bioresorbable materials, in components that include (1) an array of independent, wirelessly addressable thermal actuators, each of which consists of a resonant receiver coil connected to a serpentine resistor (Joule heating element) and (2) a uniform, multilayer coating of a thermally triggerable biological lipid membrane embedded with one or more hydrophilic drug molecules, selectively located on the Joule heating elements. Details associated with the materials and fabrication strategies appear in the Materials and methods section. Figure 1b and Supplementary Figure S2 present confocal microscope images of the layers of the lipid (green) and the drug (red) over the surface of the device. The lipid membrane consists of dipalmitoylphosphatidylcholine, 1,2-dilauroyl-sn-glycero-3-phosphoethanolamine, dipalmitoylphosphatidylglycerol or 1,2-dioleoy1-3-trimethylammonium-propane and cholesterol, specifically designed to (1) efficiently retain drugs for long periods of time *in vitro* (months), with little leakage, (2) rapidly release them upon heating beyond a critical temperature (41–43 °C) that is higher than body temperature (normal to fever; 36.5–38.5 °C) but below the maximum allowable temperature (43–45 °C) for human tissues and (3) exploit materials with established levels of biocompatibility (Food and Drug Administration-approved lipid compounds with cholesterol). This type of precise control and abrupt switching, together with separately addressable drug reservoirs, represent key advances over our recently reported silk-based systems^14^ in which diffusion of drugs occurs continuously from a single region, and the rate can be thermally accelerated.

Figure 1c schematically illustrates the two coexisting phases of the lipid membranes: liquid ordered (L_o_) and L_d_. The L_o_ phase involves rigid, all-trans tails; the L_d_ phase contains alkyl tails that can undergo rapid conformational changes.^19^ The coexistence of L_d_ and L_o_ phases is often induced by the presence of cholesterol.^20,21^ Sinha and coworkers^22^ demonstrated that the L_o_ domains segregate laterally within the lipid membrane with correlations across bilayers to form a columnar L_o_ lamellar phase that has two distinct lamellar phase repeat distances. Hydrophilic drugs bind most tightly to the head group domains. For charged drug molecules, this interaction can be enhanced by attractive Coulomb forces.^23^ Multiple-stacked lipids in the L_o_ phase hinder the permeation of the drugs.^22^ Thermal actuation induces a transition to the L_d_ phase,^24,25^ thereby allowing diffusion of the drugs out of the system. Figure 1d shows results of small angle X-ray scattering from a lipid membrane at various temperatures (*T* = 25, 40, 43 and 50 °C), with magnified views (right) near the critical temperature (*T* = 40 and 43 °C). Each reflection consists of double Bragg peaks below 40 °C (Figure 1d, blue line) that arise from the coexistence of extended L_o_ (lower *q*) and L_d_ regions (higher *q*) of the lipid bilayers. At 43 °C (Figure 1d, green line), a single Bragg peak emerges, consistent with a phase transition (from the coexistence of L_o_/L_d_ phases to the L_d_ phase) at the critical temperature between 40 and 43 °C. The even spacing of the Bragg peaks indicates a lamellar structure of regularly stacked lipid multilayers at a repeat distance *d* = 2π/*q* = 182 Å, where *q* is wave vector.^26^ Wide angle X-ray scattering (Supplementary Figure S3) measurements confirm the L_o_/L_d_ transition, where a Bragg peak characteristic for packed rigid lipid alkyl tails vanishes as the temperature exceeds the critical point.

Wireless power delivered to the Joule heating elements (for details see Supplementary Figure S4) can selectively induce this phase transition in corresponding regions of the lipid membrane. The 2 × 2 array design presented here includes four independent receiver coils with 1, 4, 6 and 9 turns, where the resulting resonant frequency is proportional to the inverse of the number of turns (Supplementary Figure S5a). Supplementary Figure S5b illustrates the phase-angle transition from inductive to capacitive impedance of the coupled coils at the peak resonant frequencies. Further improvements in coupling can be achieved by integrating capacitors to match the resonant frequencies of the transmitter to the self-resonant frequency of the receiver. Experimental results in Figure 1e show temperature distributions (FLIR SC645 infrared camera, sensitivity <0.05 °C) that follow from resonant delivery of power (0.4 W at 12 MHz) to one of the four elements in the array. Here, the temperature reaches 45 °C within 10 s, consistent with finite element analysis of the thermal response (Figure 1f). The temperature increase is spatially localized and linearly proportional to the incident power (Supplementary Figure S5c), thereby allowing controlled, independent activation of lipid associated with each element in the array. Figure 1g shows representative experimental measurements and computational (finite element analysis) results of the average temperature of the activated element (marked 4; ~ 45 °C) and adjacent elements (marked 1, 2, 3; 26–28 °C) in Figure 1e and f. The temperatures of the adjacent elements are considerably below the phase transition temperature (40–43 °C) of the lipid, thereby minimizing the possibility of off-state leakage before activation. Experimental results demonstrating that all the heating elements are capable of operating in a sequential way appear in Supplementary Figure S6. The amplification circuit used in these experiments is able to trigger the operation of only one coil at a time.

**Figure 2:**
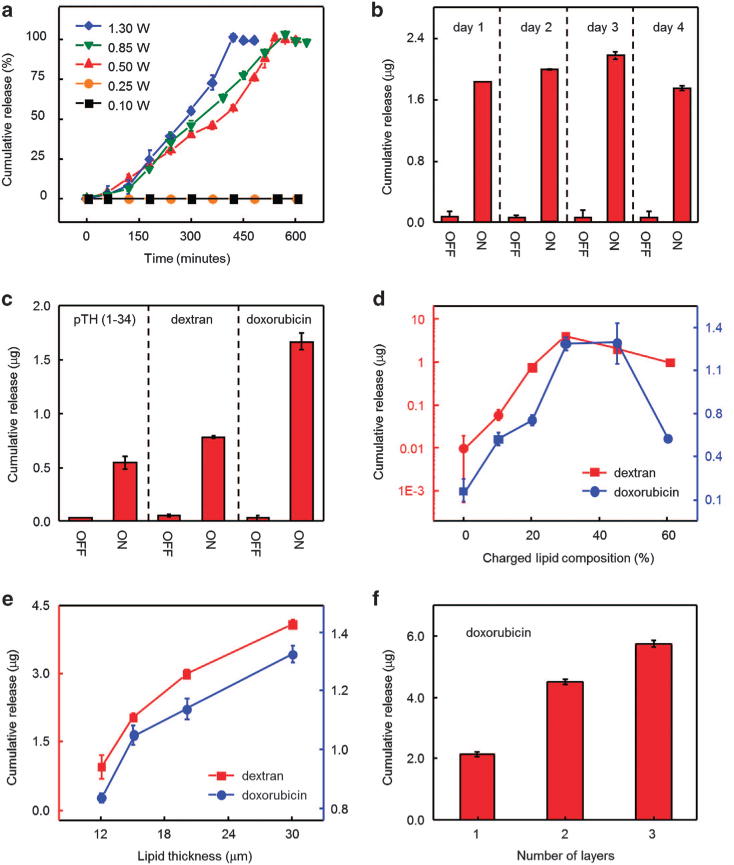
*In vitro* assessments of programmed release of drugs. (**a**) Percentage of cumulative release of doxorubicin from a device operated at wireless power levels between 0.1 and 1.3 W. (**b**) Cumulative release from a 2 × 2 array of a device at 1-day dosing cycles. The off-state leakage levels correspond to values determined 30 min before activation. (**c**) Cumulative release of parathyroid hormone (PTH(1–34)), dextran, and doxorubicin. (**d**) Cumulative release of dextran and doxorubicin for devices formed with various ratios of charged lipids. (**e**) Cumulative release of dextran and doxorubicin for devices that incorporate lipid membranes with different thicknesses. (**f**) Cumulative release of doxorubicin from devices with double and triple stacked designs.

This simple construction offers robust, wireless control of the kinetics of drug release. Figure 2a shows the cumulative release of doxorubicin, in terms of a percentage of the total, as a function of time from a device immersed in deionized water (12 ml) when activated wirelessly with externally applied power between 0.1 to 1.3 W at 12.5 MHz (four turns of a receiver coil) and a distance of 2 mm. All of the experiments in Figure 2 used deionized water to isolate the fundamental materials design principles from the chemical complexities introduced by the use of biological fluids. The results indicate linear release profiles, up to a plateau that corresponds to an average release rate of ~ 15% h^−1^. These kinetics correspond to first-order behavior, wherein the release begins abruptly at a critical temperature between 41 and 43 °C, without significant change for higher temperatures, across the range of powers examined here (Supplementary Figure S7). This observation is consistent with the thermal phase transition behavior of the cholesterol containing lipid membrane,^27^ and differs distinctly from a mechanism based on simple, temperature-dependent diffusion behavior^28^ or silk-based drug release profiles^14^ where temperature-driven annealing accerlerates the release rate of drugs by further crystallization of silk fibroin. A certain amount of leakage occurs when the power is removed before complete release of the drug, likely due to openings in the lipid membrane formed at locations where the drug molecules leave (Supplementary Figure S8).

The type of operation illustrated in Figure 2a can be useful in programmed, multi-dose drug release for the treatment of conditions that require pulsatile delivery profiles, modulated from a zero baseline. Figure 2b shows the cumulative amounts of doxorubicin released wirelessly once a day from each element in the 2 × 2 array device with externally applied power of 1.0 W at 12.5–14 MHz at a distance of 2 mm. The results exhibit well-defined, predictable responses with negligible leakage of drug during the inactivated state. Extended measurements reveal minimal leakage (< ~ 0.05 μg per day) of drug over a month yielding high on and off ratio (> ~ 50, that is, the ratio of drug released during the activation period to that released during the inactivated state) as shown in Supplementary Figure S9. Increased leakage of drug occurs in phosphate-buffered saline, as distinct from the case in deionized water due to the influence of ions in the biological fluids (Supplementary Figure S9).

Delivery of multiple different drugs, often important in clinical contexts, is also possible. Figure 2c presents experimental results that demonstrate separate, independent release of parathyroid hormone (1–34), dextran and doxorubicin by exploiting a single device (four turns of a receiver coil) with externally applied power of 1.0 W at 12.5 MHz and a distance of 2 mm. The release rates and cumulative loading of each drug vary with molecular weight (M_w_ = 4117.7, 3000.0, 579.9 for pTH (1–34), dextran and doxorubicin, respectively) as well as lipid membrane charge density. The latter affects the interaction characteristics (that is, diffusivity and Coulomb forces) of the drug molecules with the lipid membrane.^29,30^ Design of the lipid chemistry can, therefore, influence the maximum dosage. Figure 2d presents the cumulative amounts of drugs released from lipid membranes formed with different ratios by weight of charged lipid (dipalmitoylphosphatidylglycerol and 1,2-dioleoy1-3-trimethylammonium-propane for doxorubicin and dextran, respectively). Increasing the content of charged lipid strengthens the Coulomb attraction forces, thereby increasing the amount of drug that can be accommodated. The results show that the dosages for dextran and doxorubicin increase until the ratio reaches ~ 30%, followed by an abrupt decrease at higher ratios (>40%) due to aggregation of the charged lipid with the drugs.^31^ The thickness of the membrane provides another route to define the maximum dosage. Figure 2e presents, as an example, the release of controlled amounts of dextran (red dotted line) and doxorubicin (blue dotted line) from lipid membranes with thicknesses between 12 and 30 μm, achieved by selecting spin-casting speeds between 500 and 3000 r.p.m. Dosages in this case vary between 0.9–4.1 μg and 0.8–1.3 μg for dextran and doxorubicin, respectively. Loading each compartment in the array with different amounts of drugs is limited by the spin-casting process during formation of the lipid membrane. The available dose can be further increased by physically stacking devices built on thin bioresorbable supports (PLGA), as illustrated in Supplementary Figure S10. Figure 2f shows examples in double and triple layer constructions that allow two and three times the amount of drug (doxorubicin), respectively, compared with that of a single layer.

**Figure 3:**
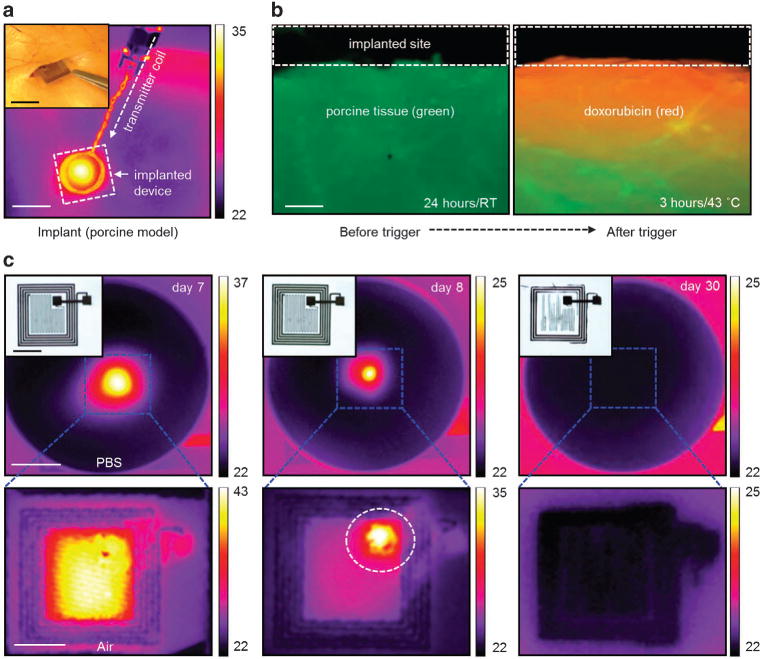
*In vivo* operation of a device in a porcine model and its *in vitro* dissolution. (**a**) Thermal maps (scale bar: 1 cm) of a device implanted in the subdermal region. Inset (scale bar: 1 cm) shows an optical image during the implantation. (**b**) Fluorescent optical image (scale bar: 300 μm) of the tissue 24 h after implantation (left) and after heating at 43 °C for 3 h (right). (**c**) Thermal maps (scale bar: 5 mm) and corresponding optical images (insets, scale bar: 1 cm) of dissolution of a device immersed in phosphate-buffered solution (PBS, pH 7.4 at 37 °C). Temperature scale bars, °C.

Practical considerations demand mechanical strength and robustness, and ability to function reliably within realistic biological environments. An experiment to demonstrate these features appears in Figure 3a, in the form of thermal images of a wirelessly activated device implanted in the subdermal region of a porcine model. (The temperatures shown in the image are smaller, by ~ 25%, compared with the device temperature due to the presence of the skin.) Figure 3b presents fluorescence microscope images that highlight drug delivered into tissue pre-colorized with a green dye (Alexa Fluor 488, Life Technologies). The results show no visible off-state leakage for 24 h after implantation (Figure 3b, left), with diffusion of the drug (red color) into the tissue only after wireless activation (Figure 3b, right). Further development of the device for long-term use *in vivo* will mitigate degradation mechanisms that arise from factors such as mechanical abrasion of the lipid membrane, and the presence of proteins, ions and enzymes.

Complete bioresorption following the programmed drug delivery process eliminates unnecessary patient risk and device load on the body. Figure 3c presents a series of thermal maps and optical images (insets) of a typical device at various times after immersion in phosphate-buffered saline solution (pH 7.4, Sigma-Aldrich) at 37 °C. The wirelessly activated increase in temperature at the surface of the solution remains almost unchanged for ~ 1 week, and then begins to rapidly decrease due to dissolution of the electrical interconnects (dashed circle in Figure 3c; middle bottom image). The addition of encapsulants based on layers of silicon oxide or silicon nitride can further extend the operational lifetimes, as studied in the context of other types of devices in previous reports.^32,33^ Complete dissolution of the Mo components (5 μm thick) occurs after 6–8 months under these conditions, consistent with previously reported dissolution rates of ~ 0.02 μm per day.^34^ The polymer layers (PLGA) used in this study possess high relative molecular masses (66 000–107 000) to provide degradation times of a few months, based on previously reported kinetics for this material.^4^ The biological lipids undergo enzymatic degradation by lipases and phospholipases, in which the kinetics strongly depend on temperature, pH, concentration of enzymes and compositions of the lipid.^35,36^ Lipid membranes that consist of compounds similar to those used here degrade only ~ 5% in 3 months, whereas ~ 95% of the encapsulated chemotherapy drug was found to be intact after 6 months when stored at room temperature in day light conditions.^37^ More additional details about bioresorption of the components, such as Mo, PLGA and lipids, of the device appear in our previous studies and others.^34–36,38^ The lipid membrane is stable throughout this timeframe, without any significant drug leakage (both in deionized water and phosphate-buffered saline media). As a result, the possibility of off-state leakage before activation is minimal, such that drug is efficiently retained before the main body of the device degrades.

**Figure 4:**
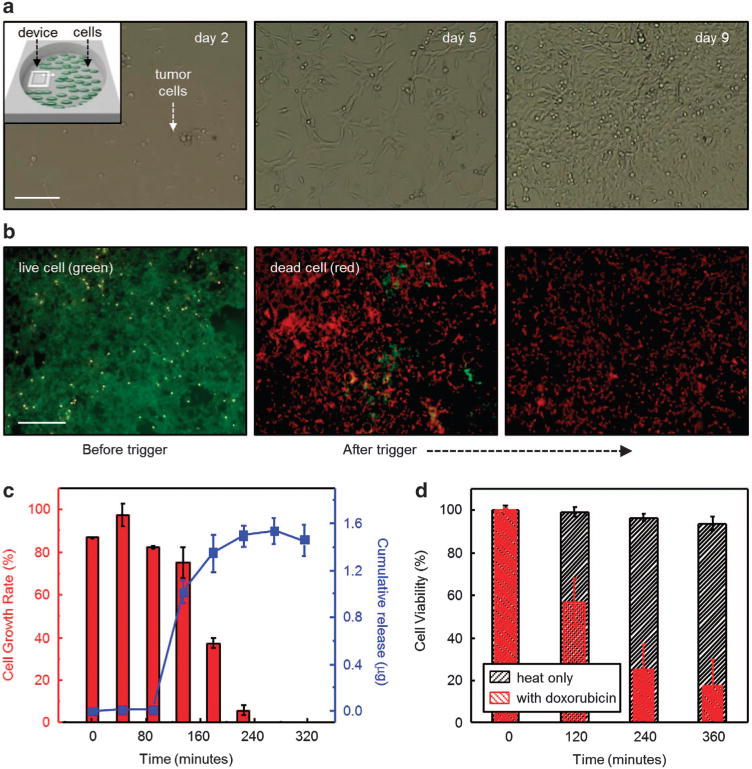
*In vitro* evaluations of a device in a proliferation/viability assay using human tumor cells (HeLa). (**a**) Optical microscope images (scale bar: 100 μm) of tumor cells at different incubation times (day 2, 5, 9 from left to right). The inset in the upper left of the left frame provides a schematic illustration of the location of the device relative to the cells. (**b**) Fluorescent optical images (scale bar: 500 μm) of the tumor cells before and after programmed release of drug. Green and red colors represent live and dead cells, respectively. (**c**) Proliferation assay and a corresponding release profile of doxorubicin. (*n* = 3, averaged data points and error bars are represented). (**d**) Viability assay using devices with (red) and without (black) doxorubicin. (*n* = 3, averaged data points and error bars are represented).

Treatment of tumor cells with doxorubicin provides an application example. Here, because different stages of cancer evolution demand different drug release rates, programmable control can improve the therapeutic efficacy and also reduce any systemic side effects, such as cardio-toxicity and congestive heart failure.^39,40^ Figure 4a presents a set of microscope images at various stages of culturing human tumor cells (HeLa) on a sterilized device loaded with doxorubicin in a cell culture dish (inset). Significant numbers of tumor cells stretch and differentiate within 5 days, to reach more than 95% confluency within 9 days, suggesting that there is negligible off-state leakage of the drug during the culturing period. Figure 4b shows fluorescence microscopy images to determine cytotoxicity using a Live/Dead Viability/Cytotoxicity kit (Life technologies), indicating that the healthy tumor cells (green dye, Calcein, Life technologies) are mostly dead (red dye, EthD-1, Life technologies) ~ 2 h after triggered release of the doxorubicin. Figure 4c and d present evaluations of proliferation and cytotoxicity of the tumor cells with drug released from a device immersed in Optimem (12 ml) via wireless heating for 5–6 h. Prior to cell exposure, the device was submerged in a separate dish containing Optimem and the drug was triggered by the temperature stimulus. The released drug solutions were collected at specific time points and transferred to cell culture 96-well plates. The results show that the growth rate and the viability of the tumor cells decrease most significantly within the first 2–3 h after triggered release, followed by gradual reduction to subtle levels (0% and <20% for the cell growth rate and the viability, respectively). A representative release profile appears in Figure 4c (blue dotted line). Control studies showing that the device remains stable without any drug release during the course of the cell culture experiments appear in Supplementary Figure S11. These results indicate that cancer cell growth suppression is not associated with passive leakage but, instead, by actively triggered released drugs. Other control experiments performed without the drug suggest that the increases in temperature alone are insufficient (<10%) to account for the observed cytotoxicity, as shown in Figure 4d (black dashed bar). Details associated with the cell culturing procedures and treatments appear in the Materials and methods section.

**Figure 5:**
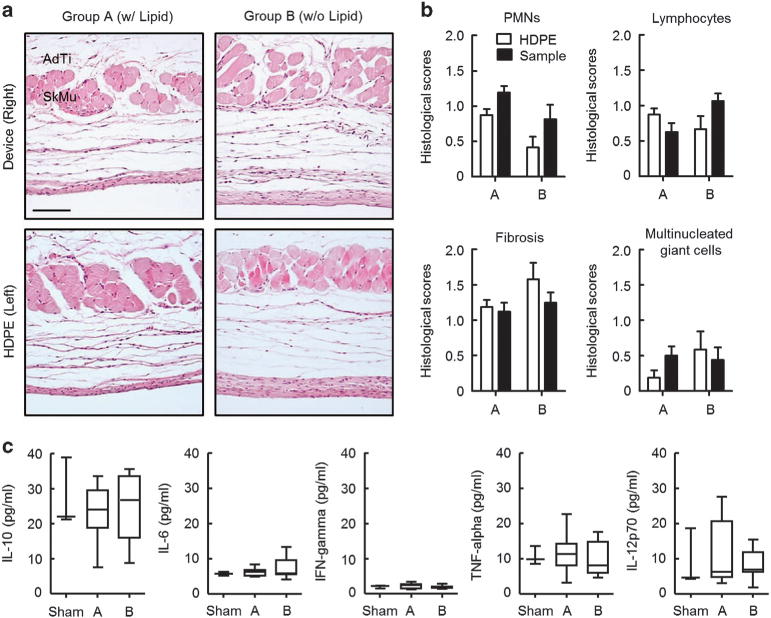
*In vivo* biocompatibility studies. (**a**) Representative microscope images (scale bar: 100 μm) of hematoxylin and eosin (H&E) stained tissue sections at 5 weeks post-implantation. (**b**) Evaluations of the histological score were performed in over 5 randomly chosen high-power fields in reference controls (HDPE) and test groups (means ± s.e.m., *n* = 5 per group). (**c**) Measured levels of inflammatory cytokines in peripheral blood serum by cytometric bead arrays (CBA). Statistical significance was determined by one-way analysis of variance (ANOVA) followed by Bonferroni’s multiple comparison test. Significance was ascribed at *P*<0.05. AdTi, adipose tissue; HDPE, high-density polyethylene; IFN-γ, interferon-γ; IL-10, interleukin-10; PMN, polymorphonuclear; SkMu, skeletal muscle; TNF-α, tumor necrosis factor.

*In vivo* biocompatibility is a critical consideration for the achievement of long-term device engraftment. To test the biocompatibility of our devices, Balb/c mice (*n* = 10) were implanted at the right side of subcutaneous pockets with device containing lipid (Group A) or device without the lipid (Group B) in accordance with Institutional Animal Care and Use Committee protocols (Supplementary Figure S12a). The same mice also underwent the surgical procedure on the left side of subcutaneous pockets with non-toxic HDPE (Food and Drug Administration approved) controls to provide a baseline tissue inflammation caused by implantation. In addition, a group of mice were subjected to the same implantation procedure but with no device or materials, to provide a baseline for comparison (Sham, shown in Supplementary Figure S12b). After sagittal skin incisions with sterilized clips, the mice were returned to the specific pathogen-free facility until analysis. As compared with the sham-operated (that is, no implant) control group, mice with implanted devices exhibit normal behaviors with no significant body weight loss during implantation period of 5 weeks (Supplementary Figure S12c). Hematoxylin and eosin staining and examination of the local cytotoxicity of skin sections demonstrate comparable levels of immune cells including polymorphonuclear cells, lymphocytes and multinucleated giant cells, compared with the control groups (Figure 5a and Supplementary Figure S13). A histological analysis of the biocompatibility at 5 weeks post-implantation appears in Figure 5b and Supplementary Table S1.

Measurements of the level of inflammatory cytokines and the percentages of immune cells in the blood allow assessment of systemic immune reactions over a period of 5 weeks (Figure 5c). The levels of IL-10, IL-6, interferon-γ, tumor necrosis factor-α and IL-12p70 are comparable to those of the control group, suggesting that the implanted devices induce no inflammatory responses. In addition, there are no abnormal changes in the populations of CD4+ T cells, CD8+ T cells, B cells, NK cells, neutrophils, monocytes and macrophages in peripheral blood (Supplementary Figure S14) along with those in the spleen by comparisons between the control and the test groups (Supplementary Figure S15). Taken together, these findings suggest that the devices can be considered as biocomparable temporary implants for applications in drug delivery.

## CONCLUSION

The results presented here demonstrate that bioresorbable wireless electronics can be combined with thermally activated lipids for remotely controlled release of drugs in a time sequenced manner, with full, programmable rate kinetics from values that are near zero to those that can be set by choice of lipid chemistry and structure. The materials, device designs and fabrication strategies for these platforms offer an expanded set of options in drug delivery, with potential to improve patient compliance and the efficacy of current clinical procedures. Deep tissues can be addressed by using near-surface coils connected by bioresorbable wires to the implant site. Although the results focus on advantages provided by lipid-based layered films, other material systems, such as those based on hydrogels can be considered.

## Supplementary Material

Supplemental
