# Supplementary material for: Biological lipid membranes for on-demand, wireless drug delivery from thin, bioresorbable electronic implants
Source: NPG Asia Mater. Author manuscript; Available in PMC 2016 Nov 1. (PMC4861403; doi:10.1038/am.2015.114)
Supplement: Supplemental [file NIHMS778786-supplement-Supplemental.pdf]

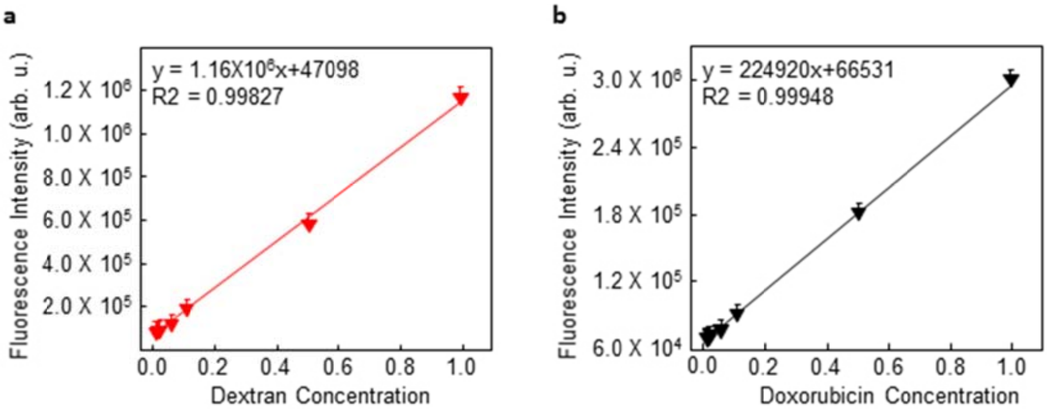
a
y = 1.16X10^6x+47098
R2 = 0.99827
1.2 X 10^6
1.0 X 10^6
8.0 X 10^5
6.0 X 10^5
4.0 X 10^5
2.0 X 10^5
Fluorescence Intensity (arb. u.)
0.0
0.2
0.4
0.6
0.8
1.0
Dextran Concentration
b
y = 224920x+66531
R2 = 0.99948
3.0 X 10^5
2.4 X 10^5
1.8 X 10^5
1.2 X 10^5
6.0 X 10^4
Fluorescence Intensity (arb. u.)
0.0
0.2
0.4
0.6
0.8
1.0
Doxorubicin Concentration

FIGURE S1

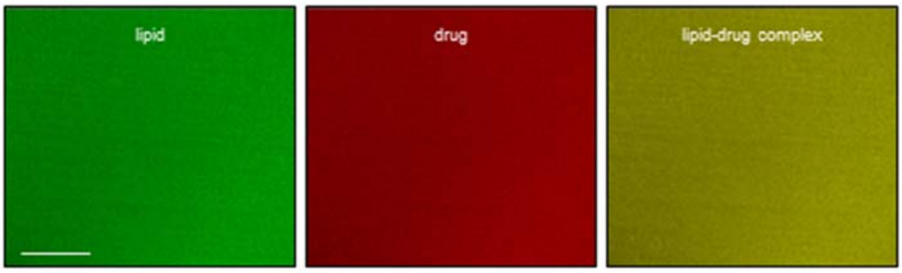

FIGURE S2

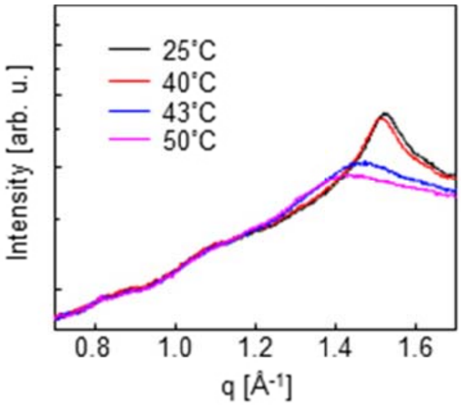

FIGURE S3

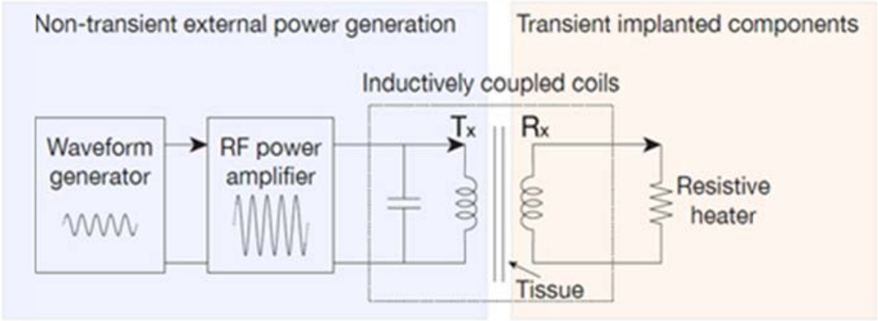
Non-transient external power generation
Transient implanted components
Inductively coupled coils
Waveform generator
RF power amplifier
Tx
Rx
Resistive heater
Tissue

FIGURE S4

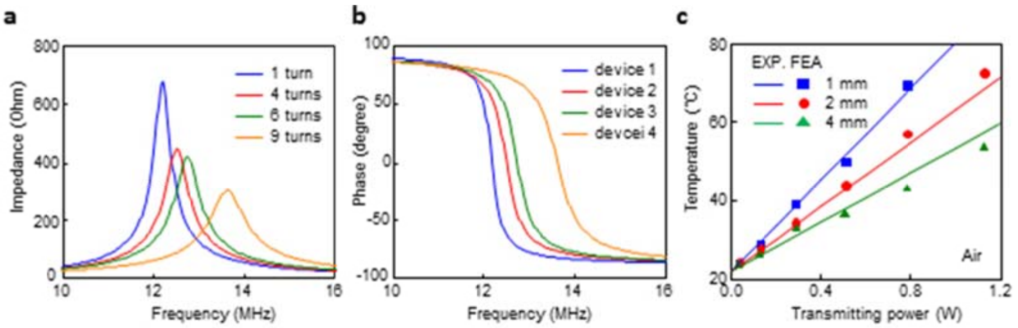
a
b
c
Impedance (Ohm)
Frequency (MHz)
1 turn
4 turns
6 turns
9 turns
Phase (degree)
device 1
device 2
device 3
devcei 4
Temperature (℃)
Transmitting power (W)
EXP. FEA
1 mm
2 mm
4 mm
Air

FIGURE S5

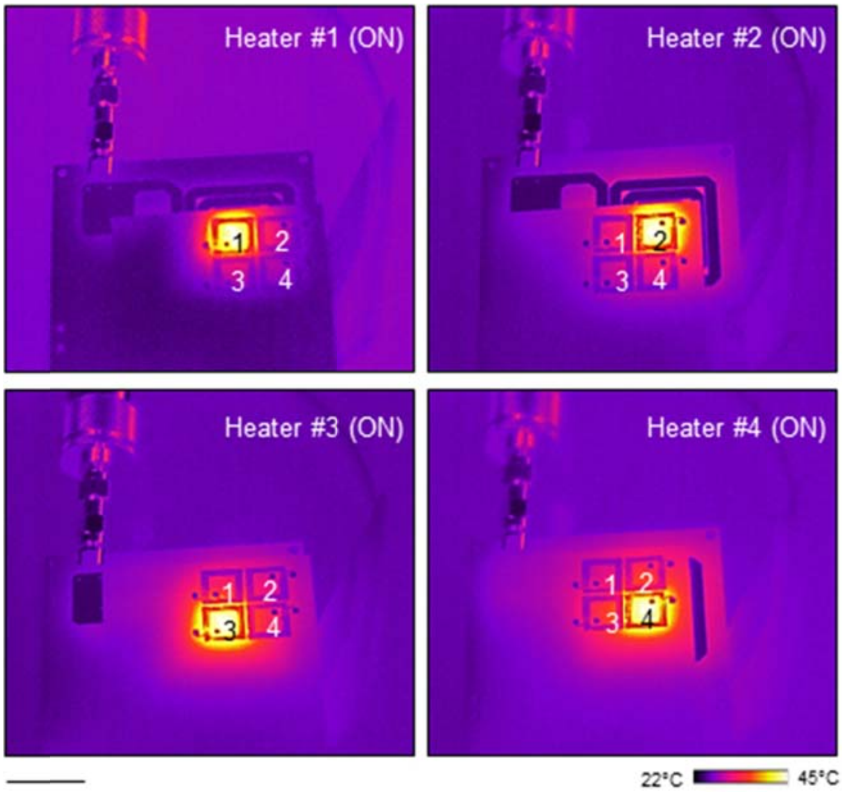

FIGURE S6

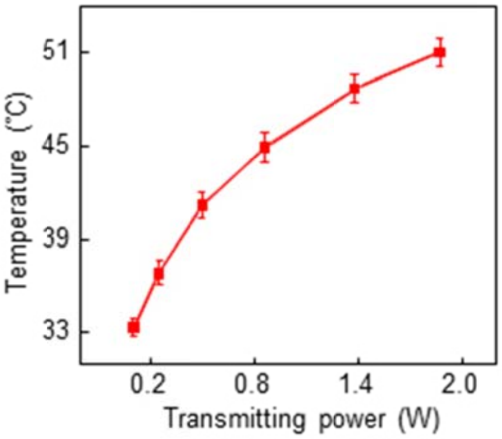

FIGURE S7

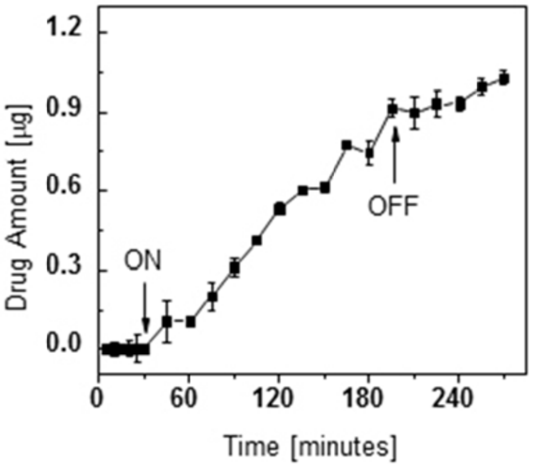
1.2
0.9
0.6
0.3
0.0
Drug Amount [µg]
ON
OFF
0
60
120
180
240
Time [minutes]

FIGURE S8

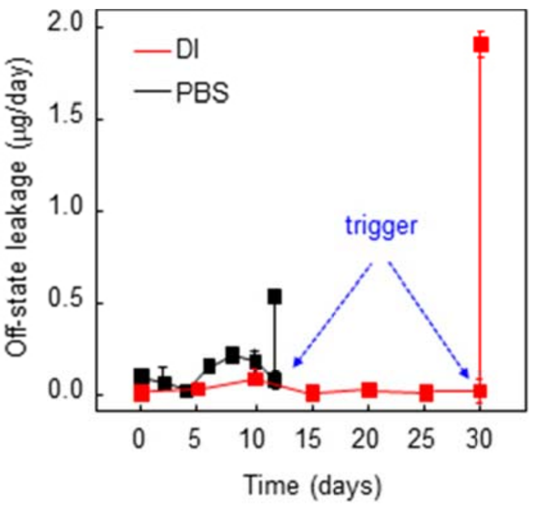

DI
PBS
trigger
Off-state leakage (μg/day)
Time (days)
2.0
1.5
1.0
0.5
0.0
0
5
10
15
20
25
30

FIGURE S9

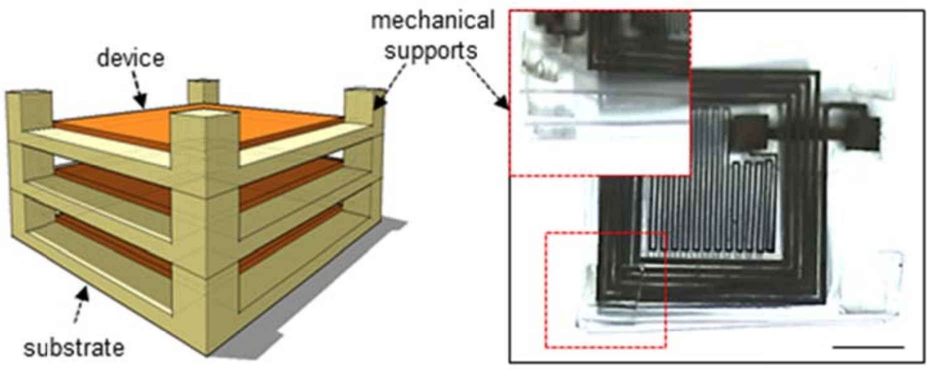

FIGURE S10

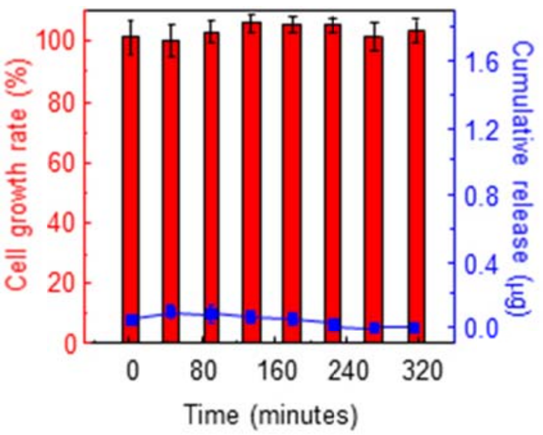

Cell growth rate (%)
100
80
60
40
20
0
0
80
160
240
320
Time (minutes)
1.6
1.2
0.8
0.4
0.0
Cumulative release (μg)

FIGURE S11

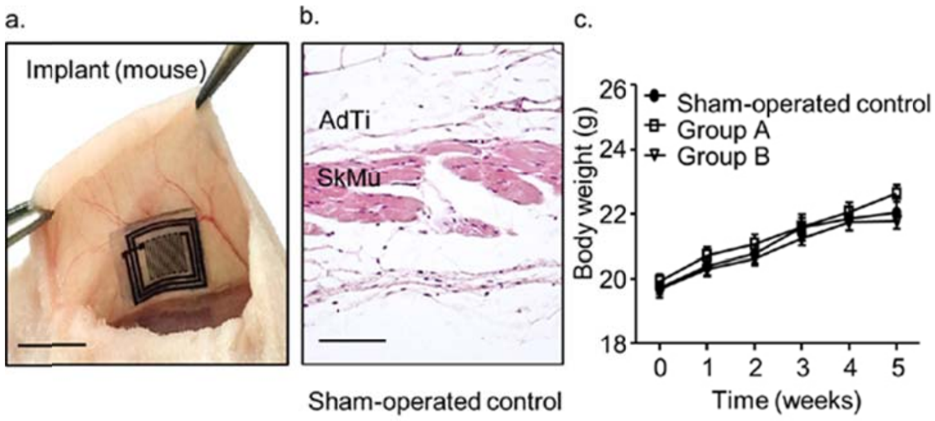
a.
Implant (mouse)
b.
AdTi
SkMu
Sham-operated control
c.
Sham-operated control
Group A
Group B
Body weight (g)
Time (weeks)
26
24
22
20
18
0
1
2
3
4
5

FIGURE S12

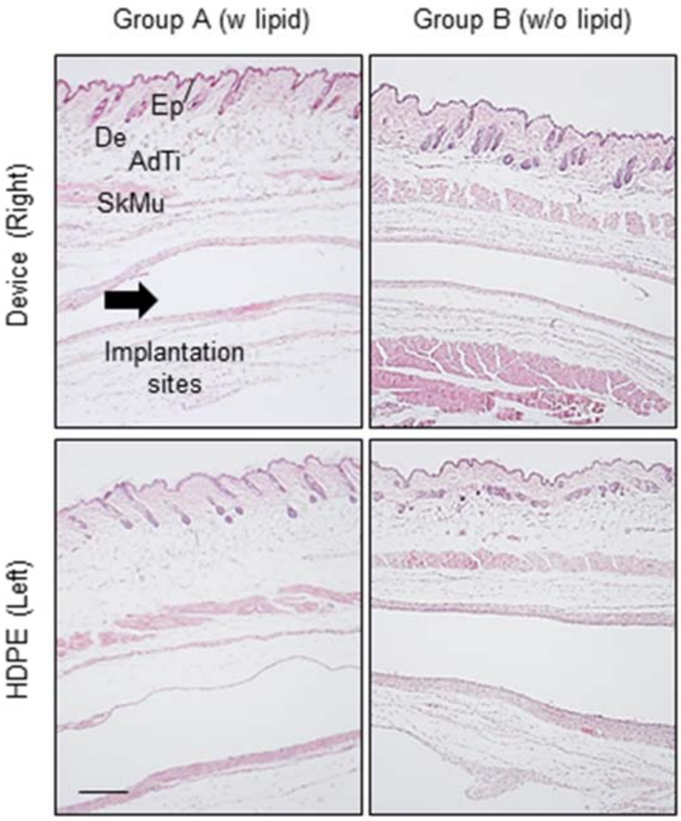
Group A (w lipid)
Group B (w/o lipid)
Device (Right)
HDPE (Left)
Ep
De
AdTi
SkMu
Implantation sites

FIGURE S13

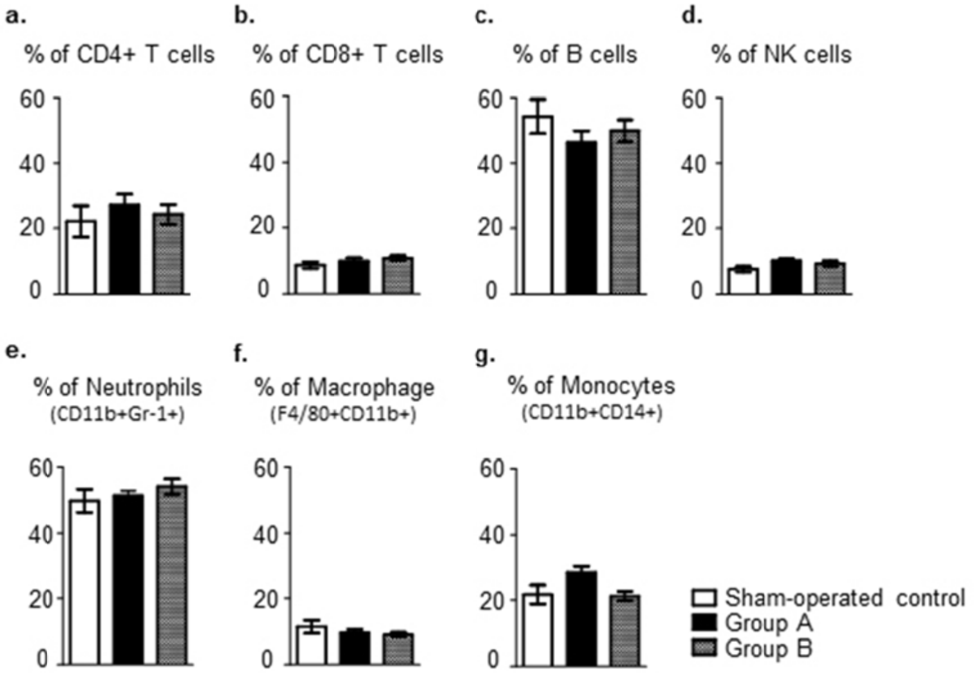

a.
% of CD4+ T cells
b.
% of CD8+ T cells
c.
% of B cells
d.
% of NK cells
e.
% of Neutrophils
(CD11b+Gr-1+)
f.
% of Macrophage
(F4/80+CD11b+)
g.
% of Monocytes
(CD11b+CD14+)
60
40
20
0
Sham-operated control
Group A
Group B

FIGURE S14

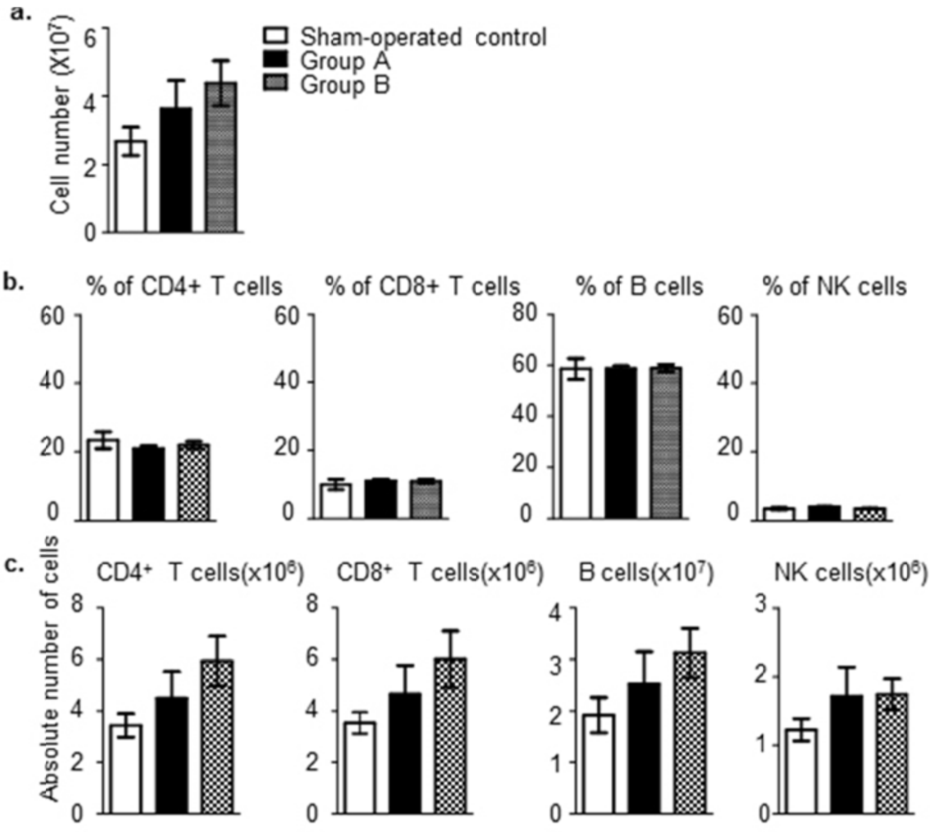
a.
Cell number (X10^7)
Sham-operated control
Group A
Group B
b.
% of CD4+ T cells
% of CD8+ T cells
% of B cells
% of NK cells
c.
Absolute number of cells
CD4+ T cells(x10^6)
CD8+ T cells(x10^6)
B cells(x10^7)
NK cells(x10^6)

FIGURE S15

Implant		PMCs[a]	Lymphocytes[b]	Multinucleated giant cells[c]	Neovasculatisation[d]	Fibrosis[e]
Sham-operation control			-	-	-	-
A	HDPE	0.88 ± 0.34	0.88 ± 0.34	0.19 ± 0.4	0.75 ± 0.58	1.19 ± 0.4
	sample	1.19 ± 0.4	0.63 ± 0.5	0.5 ± 0.52	0.75 ± 0.45	1.13 ± 0.5
B	HDPE	0.42 ± 0.51	0.67 ± 0.65	0.58 ± 0.9	1 ± 0.74	1.58 ± 0.79
	sample	0.81 ± 0.83	1.06 ± 0.44	0.44 ± 0.73	0.94 ± 0.57	1.25 ± 0.58

[a] Polymorphonuclear cells: 1-5/phf* (1), 5-10/phf(2), >10/phf (3)

[b] Lymphocytes: 1-5/phf (1), 5-10/phf(2), >10/phf (3), >10/phf (3)

[c] Multinucleated giant cells: 1-5/phf (1), 5-10/phf(2), >10/phf (3)

[d] Neovascularisation: Minimal capillary proliferation (1), Groups of 4-7 capillaries (2), Broad band of capillaries (3)

[e] Fibrosis: Narrow band (1), Moderately thick band (2), Thick band (3)

* phf = per high powered (400 ×) field.

SI FIGURE LEGENDS

Figure S1 Standard curve for drug concentration calculation. Fluorescence intensity and drug concentration are linearly proportional from 0.001 g/ml to 1 g/ml for (**a**) dextran and (**b**) doxorubicin.

Figure S2 Two dimensional (2D) fluorescent optical images (scale bar: 500 μm) of lipid membrane (green), loaded drug (red) and their overlapped image (yellow).

Figure S3 Transmission Wide Ange X-ray Scattering (WAXS) scans of the lipid membrane at various temperatures.

Figure S4 Schematic diagram of the wireless power delivery system. A signal generator generates, amplifies, and delivers AC waveforms to a transmitter coil while an inductive coupling receiver coil receives these waveforms and delivers the power to a resistive heating element.

Figure S5 (**a, b**) Measured impedance and phase of the coupled transmitter and receiver for frequencies between 10 to 16 MHz. (**c**) Experimental (lines) and FEA (dots) results corresponding to changes in local temperature of an activating heater as a function of transmitter

driving power at different separations (1, 2, 4 mm) between the primary coil and the device, defined by the thickness of a piece of porcine tissue.

Figure S6 Collected infrared (IR) image (scale bar: 2 cm) during operation of all heating element in the 2×2 array device sequentially in a clockwise direction.

Figure S7 Doxorubicin release profile showing continued elution of drug (doxolubicin) after turned off the power early before the drug is completely released.

Figure S8 Changes in local temperature at the surface of an activated heater in phosphate buffer solution (PBS, 6 mL) as a function of transmitting power between 0.2 and 2.0 W.

Figure S9 Measured off-state leakage of doxorubicin from a lipid membrane laminated on a non-biodissolvable substrate (aluminium foil) over a month in deionized water (red line) and 12 days in PBS (black line).

Figure S10 Schematic illustration (left) and optical image (right) of a triple stacked device using mechanical supports made of PLGA. Inset (scale bar: 3 mm) shows an enlarge image at the edge.

Figure S11 Proliferation assay of the drug from the untriggered device and corresponding doxorubicin release profile showing leaked amount of drug has a negligible effect on cancer cell growth suppression. (n = 3, averaged data points and error bars are represented)

Figure S12 (**a**) Optical image (scale bar: 1cm) of subcutaneous implantation of a device into mice. (b) Histological examination (Scale bar: 100 μm) of tissue sections in sham-operated control. Images of paraffin sections stained with Hematoxylin & eosin. SkMu; Skeletal muscle, AdTi; Adipose tissue. (**c**) Measurement of body weight of mice implanted with a device. Data are represented mean ± SEM.

Figure S13 Histological examination (Scale bar: 100 μm) of tissue sections in sham-operated control. Images of paraffin sections stained with Hematoxylin & eosin. SkMu; Skeletal muscle, AdTi; Adipose tissue.

Figure S14 Immuno-profiling of lymphocytes from peripheral bloods at 5 weeks post-implantation of group A, B and operated-control groups. (**a**) CD4+ T cells (CD3+CD4+), (**b**) CD8+ T cells (CD3+CD8+), (**c**) B cells (CD3-CD19+), (**d**) NK cells (CD3-NK1.1+), (**e**) Neutrophils (CD11b+Gr-1+), (**f**) Macrophage (F4/80+CD11b+), and (**g**) Monocytes (CD11b+CD14+) population are presented as percentages of total PBMCs in the peripheral blood by flow cytometry. (n = 10, mean ± SEM)

Figure S15 Flow cytometric analysis of splenocytes from mice implanted with group A, B devices and sham-operated control at 5 weeks post-implantation. (**a**) Total number of splenocytes following sham-operated and two test groups. (**b**) Percentage of indicated cell populations using flow cytometry. (**c**) Absolute number of various immune cell subsets in spleen. Data presented are the means ± SEM.

Table S1 Histological quantitative analysis of biocompatibility at five weeks post-implantation of group A and B materials. Randomly selected three fields per slide were counted at ×400 magnification. Inflammatory cells (polymorphonuclear cells, lymphocytes, and multinucleated giant cells) infiltration were scored as described. Values are given as means ± SEM.
